# Primary pulmonary follicular lymphoma

**DOI:** 10.1093/jscr/rjae730

**Published:** 2024-11-25

**Authors:** Sarah E Kim, Daniel Steeno, Alexander P Lynch, Francis J Podbielski

**Affiliations:** Department of Surgery, University of Illinois at Chicago, 840 S. Wood Street, Chicago, IL 60612, United States; Department of Surgery, University of Illinois at Chicago, 840 S. Wood Street, Chicago, IL 60612, United States; Division of Biological Sciences, Creighton University, 2500 California Plaza, Omaha, NE 68178, United States; Department of Thoracic Surgery, Jesse Brown Veterans Affair Medical Center, 820 S. Damen Avenue, Chicago, IL 60612, United States

**Keywords:** follicular lymphoma, primary pulmonary lymphoma, thoracotomy, non-Hodgkin’s lymphoma, operative resection

## Abstract

Primary pulmonary follicular lymphoma is an extremely rare subset of extra-nodal non-Hodgkin’s lymphoma. We present a successful work-up and surgical management of this disease entity. The patient is a 74-year-old man who presented with an enlarging ground glass opacity on his computed tomography scan. Percutaneous biopsy was not diagnostic for malignancy, but given the underlying malignant potential, he underwent definitive operative resection rather than additional invasive diagnostic testing. Our case highlights challenges in the management of nondiagnostic preliminary pathology as well as the role of a multidisciplinary approach to treatment of a rare lung pathology.

## Introduction

Primary pulmonary lymphomas (PPL) represent only 3%–4% of extranodal non-Hodgkin’s lymphoma and <0.5%–1% of primary pulmonary malignancies [1]. We present a case with the finding of a primary pulmonary follicular lymphoma. Our patient presented with an enlarging ground glass opacity in the right upper lobe of the lung that had been followed with serial imaging for over 2 years; he ultimately underwent a surgical resection after a nondiagnostic pathologic result from a CT-guided biopsy.

## Case report

A 74-year-old man with medical history notable for long-term tobacco use and prostate cancer status post radical prostatectomy and radiation who was found on CT scan to have a ground glass opacity measuring 1.2 cm in his right upper lobe of his lung. Further imaging with positron emission tomography (PET)/CT scan showed an elevated standardized uptake at the site of the right upper lung nodule. A CT-guided biopsy showed atypical cells, however, was not diagnostic for malignancy. Due to the possibility of underlying malignancy, the patient elected to undergo operative resection.

He underwent a right posterolateral thoracotomy, right upper lobe wedge resection, and mediastinal node biopsy. Upon entering the chest, the patient was found to have several areas of filamentous adhesions that were divided. The inferior pulmonary ligament and the pleural reflection around the hilum were divided, and the lymphatic nodal tissue was sampled. The nodule was readily palpable in the posterior segment of the upper lobe, and a stapled wedge resection was performed. Gross examination of the specimen in the operating room revealed a well-circumscribed nodule from which material was collected for culture. A section of a wedge resection along with the nodule was sent to pathology for a frozen section and showed only lymphoid tissue without evidence of gross malignancy. Palpation of the remainder of the lungs did not yield any additional suspicious nodules. The patient was extubated in the operating room having tolerated the procedure well and discharged after several days after an uneventful postoperative course.

**Figure 1:**
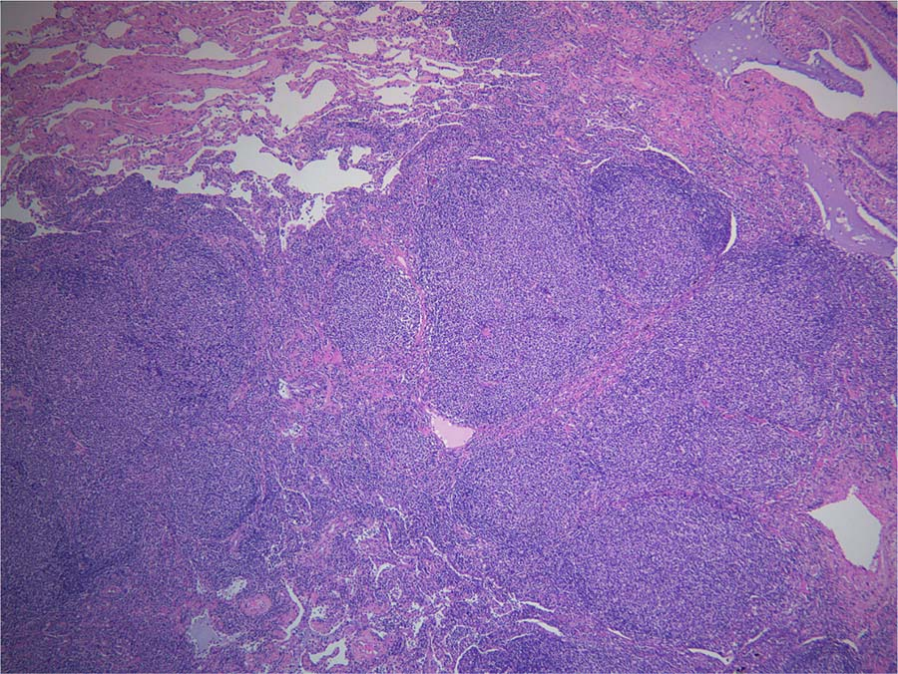
Hematoxylin and eosin. Demonstrates back to back neoplastic follicles within lung tissue.

**Figure 2:**
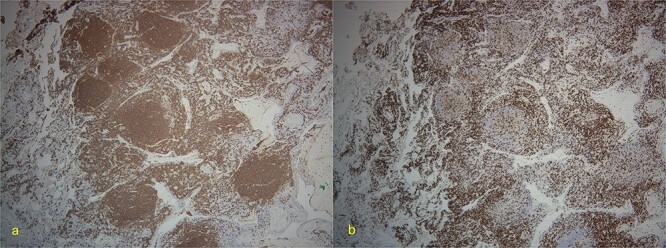
(a) 4×-Immunostain for CD20 highlights B-cells in neoplastic follicles. (b) CD3 highlights reactive T-cells around neoplastic follicles.

Final pathology with immunostaining ultimately revealed a follicular lymphoma. Figure 1 highlights the neoplastic follicles within the lung tissue with decreased macrophage bodies. Immunostains for CD 20 and CD3 highlighted B cells and reactive T cells around the neoplastic follicles (Fig. 2a and b). Other immunohistochemistry findings were notable for negative BCL2 and CD10. Molecular fluorescence in situ hybridization testing also detected BCL6 gene rearrangements. Overall, the histology and immunohistochemical findings are diagnostic of atypical follicular process combined with monoclonal IgH gene and BCL6 positive gene rearrangements, consistent with follicular lymphoma.

The hematology/oncology service was consulted given pathology findings of follicular lymphoma, with plans for post-operative surveillance laboratory testing every 3–6 months for 5 years and then annually or as clinically indicated. He was also recommended to undergo surveillance CT scans every 6 months for 5 years, followed by annual surveillance CT imaging.

## Discussion

PPL is currently defined as a clonal lymphoid proliferation affecting the lungs without any detectable extrapulmonary involvement from diagnosis to 3 months after diagnosis [1]. A majority (nearly 58%–87%) of these cases are low-grade non-Hodgkin’s lymphoma with 90% of these cases corresponding to the MALT type [2]. Follicular subtypes are thought to have many similar clinical and radiographic findings as the MALT type and thus are often difficult to differentiate from follicular lymphoma from the MALT type.

Many patients with low-grade PPL are often asymptomatic at the time of diagnosis with the disease process identified solely by radiographic findings. Common findings include a localized alveolar opacity with a diameter of <5 cm. Computed tomography is more sensitive; however, adequate tissue sampling is required for diagnosis. Bronchoalveolar lavage can be particularly useful if findings yield lymphocytic alveolitis and can be considered a specific sign when there is >10% of B lymphocytes [3]. The usual manner of diagnosis is performing immunohistochemical chemical analysis on tissue sampling. Common histological findings include absence of tangible body macrophages and positive immunostains for CD20, BCL2, CD 10, CD19, CD22, BCL6, CD21, and CD23 [4]. Additionally, extrapulmonary nodal extension is ruled out with PET CT imaging.

Current treatment options include surgery, chemotherapy, and radiation. Surgery is often preferred for localized treatment, with chemotherapy being reserved for bilateral or extrapulmonary involvement, progression, or relapse. Radiation is rarely used but can be considered for local control.

In conclusion, the lung is a common site of secondary involvement of non-Hodgkin lymphoma; however, PPLs are rare, especially the follicular subtype. This case with a rare pathologic subtype of lymphoma illustrates the importance of preoperative workup of primary lung nodules, but especially the need to consider early operative intervention rather than prolonged surveillance imaging even in the setting of nondiagnostic initial pathology in patients with higher risks of malignancy. Given this elevated risk, we elected to proceed with not only an excisional surgical biopsy of the suspicious nodule, but additionally performing a mediastinal lymph node dissection to ensure the disease was truly solitary in nature. This patient was found to have a rare extra-nodal primary pulmonary follicular lymphoma, which generally suggests an excellent prognosis with complete surgical extirpation and careful surveillance imaging.

## References

[1] Cadranel J , Wislez M, Antoine M. Primary pulmonary lymphoma. Eur Respir J 2002;20:750–62. 10.1183/09031936.02.00404102. 12358356

[2] Fiche M , Capron F, Berger F, et al. Primary pulmonary non-Hodgkin’s lymphomas. Histopathology 1995;26:529–37. 10.1111/j.1365-2559.1995.tb00271.x. 7665143

[3] Betsuyaku T , Munakata M, Yamaguchi E, et al. Establishing diagnosis of pulmonary malignant lymphoma by gene rearrangement analysis of lymphocytes in bronchoalveolar lavage fluid. Am J Respir Crit Care Med 1994;149:526–9. 10.1164/ajrccm.149.2.8306056. 8306056

[4] Kurz KS , Kalmbach S, Ott M, et al. Follicular lymphoma in the 5th edition of the WHO-classification of haematolymphoid neoplasms—updated classification and new biological data. Cancer 2023;15:785. 10.3390/cancers15030785. PMC9913816 36765742

